# Cyborg Moth Flight Control Based on Fuzzy Deep Learning

**DOI:** 10.3390/mi13040611

**Published:** 2022-04-13

**Authors:** Xiao Yang, Xun-Lin Jiang, Zheng-Lian Su, Ben Wang

**Affiliations:** 1 School of Information Science and Technology, Hangzhou Normal University, Hangzhou 311121, China; 20170056@hznu.edu.cn; 2 Department of Engineering Technology and Application, Army Infantry College, Nanchang 330100, China; xulinjiang@163.com; 3 College of Field Engineering, Army Engineering University, Nanjing 210007, China; suzhenglian@compintell.cn

**Keywords:** intelligent cyborgs, cyborg moth, deep learning, fuzzy systems, flight control

## Abstract

Cyborg insect control methods can be divided into invasive methods and noninvasive methods. Compared to invasive methods, noninvasive methods are much easier to implement, but they are sensitive to complex and highly uncertain environments, for which classical control methods often have low control accuracy. In this paper, we present a noninvasive approach for cyborg moths stimulated by noninvasive ultraviolet (UV) rays. We propose a fuzzy deep learning method for cyborg moth flight control, which consists of a *Behavior Learner* and a *Control Learner*. The *Behavior Learner* is further divided into three hierarchies for learning the species’ common behaviors, group-specific behaviors, and individual-specific behaviors step by step to produce the expected flight parameters. The *Control Learner* learns how to set UV ray stimulation to make a moth exhibit the expected flight behaviors. Both the *Control Learner* and *Behavior Learner* (including its sub-learners) are constructed using a Pythagorean fuzzy denoising autoencoder model. Experimental results demonstrate that the proposed approach achieves significant performance advantages over the state-of-the-art approaches and obtains a high control success rate of over 83% for flight parameter control.

## 1. Introduction

Cyborg insects, i.e., insect–computer hybrid robots that combine living insects and miniature machines, have numerous potential applications including search and rescue, target detection and surveillance, network fault location and maintenance, animal population control in agriculture, and endangered species protection, to name just a few [1,2,3,4]. This new field introduces the possibility, beyond traditional bio-inspiration, of the merging of natural and artificial worlds in synergistic systems [5]. The study of cyborg insects, in its modern form, started with the pioneering work of Holzer and Shimoyama [6], who built a line-tracing electronic backpack that obtains input from two photosensors and uses an on-board algorithm to control cockroaches (*Periplaneta Americana*) via electric stimulation to walk along a black line. The locomotion control of cockroaches has also been realized by stimulation of ganglia [7], antennae, and cerci [8,9]. Recently, more studies have been devoted to flying cyborg insects such as beetles [10,11,12,13], moths [14,15,16], locusts [17,18], and bees [19,20], which are mainly achieved by electrical stimulation of nervous systems, especially the efferent nerves related to the flying muscles [21].

All the above studies develop cyborg insects based on invasive stimulation methods, which require delicate microsurgery skills to accurately place electronics in insect tissues, where the resultant injury has the potential to affect flight performance [14]. Another important consideration is the public acceptance of cyborg insects, in particular, perceptions of whether insects experience pain during implantation surgeries and stimulations [22]. Therefore, noninvasive stimulation represents the best path for developing highly efficient systems as well as encouraging public acceptance from an ethical point of view [5]. However, current studies on noninvasive cyborg insects are still few. Zheng et al. [23] proposed a noninvasive method that uses an online light-emitting diode (LED) display system to present visual stimulus within ultra-low latency to induce bumblebee flight behaviors, which employs reinforcement learning coupled with sequential *K*-means clustering to generate an optimal control sequence. In [24], Zheng et al. modeled tethered bumblebee fight control as a finite and deterministic Markov decision process, and they employed Sarsa with a transformed reward function to learn the optimal control policy. The results demonstrated that the noninvasive methods can also ensure satisfactory control performance while avoiding implantation to insect tissues and reducing physical injury. Nevertheless, compared to implanted methods, noninvasive methods not only have higher sensitivity to noise and lower control accuracy but also require more extensive experimental data for training. Consequently, there are few reports of cyborg insects with noninvasive stimulation outside of the laboratory with highly controlled conditions.

To address the above difficulties, this paper proposes a fuzzy deep learning approach to the flight control of moths *Fusarium camelliae* stimulated by ultraviolet (UV) rays. The control model consists of a *Behavior Learner* and a *Control Learner*. The *Behavior Learner* is further divided into three hierarchies: (1) the lower hierarchy of layers for learning the species’ common behaviors in response to external stimuli; (2) the middle hierarchy of layers for learning the specific behaviors of different groups of moths, where the grouping is performed by a fuzzy clustering layer; and (3) the upper hierarchy of layers for learning the specific behaviors of each individual moth. The *Control Learner* learns how to set UV ray stimulation to make a moth exhibit the expected flight behaviors. The *Control Learner* and the sub-learners of the *Behavior Learner* are all constructed using a fuzzy deep learning model. Experimental results demonstrate that the proposed approach achieves significant performance advantages over other popular methods. The main contributions of this paper are as follows:

- We present a noninvasive cyborg moth design approach based on fuzzy deep learning for flight control;
- We propose a novel hierarchical fuzzy deep learning model that effectively learns the species common behaviors, group-specific behaviors, and individual-specific behaviors to achieve a high control success rate.
- We propose a new fuzzy clustering method based on Pythagorean-type fuzzy sets for moth grouping.
- The proposed approach can be easily extended for behavior learning of other cyborg animals and, therefore, contributes to the development of biobots.

In the rest of this paper, Section 2 presents the fuzzy deep learning model, Section 3 and Section 4 describe the *Behavior Learner* and *Control Learner* in detail, respectively, Section 5 presents the experimental results, and Section 6 concludes with a discussion.

## 2. Overview of the Model for Cyborg Flight Control

### 2.1. Model Architecture

**Figure 1:**
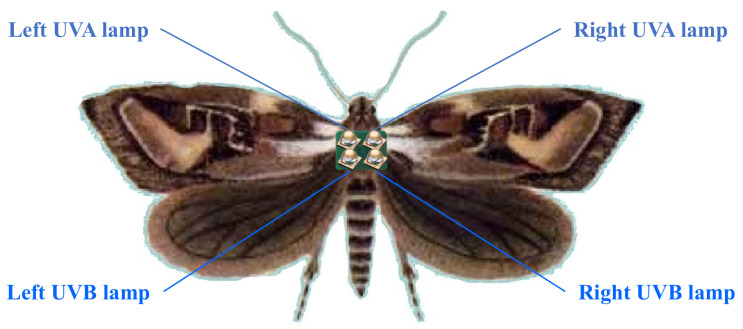
Illustration of a cyborg moth equipped with a backpack chip.

In our study, a cyborg moth is equipped with a wireless backpack chip, which has four micro UV LED lamps, including a UVA radiation lamp and a UVB radiation lamp in each of the left and right sides, as illustrated in Figure 1. The cyborg is designed to use UV ray stimulation to control the flight of the moth in a three-dimensional (3D) space. The control model outputs seven flight parameters, including the horizontal deflection angle $\theta_{h}$, the horizontal angular velocity $\varpi_{h}$, the vertical deflection angle $\theta_{v}$, the vertical angular velocity $\varpi_{v}$, and the accelerations in the *x*-, *y*-, and *z*-axes, denoted by $a_{x}$, $a_{y}$, and $a_{z}$, respectively.

**Figure 2:**
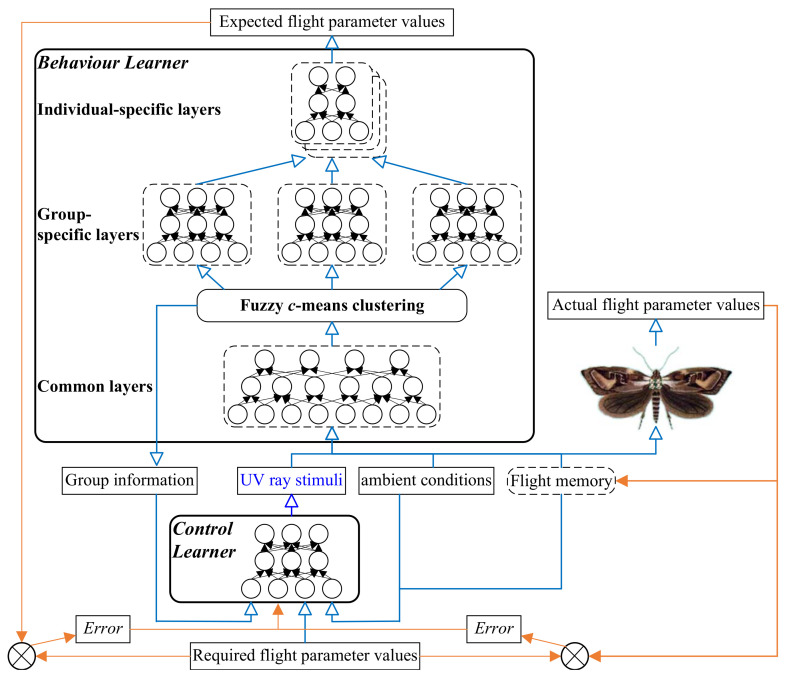
The framework of the fuzzy deep learning model for cyborg control. The *Control Learner* and the three sub-learners of the *Behavior Learner* are all constructed using the proposed PFDDAE model.

The proposed control model consists of a *Behavior Learner* and a *Control Learner*. As shown in Figure 2, the *Behavior Learner* is used to learn, under a given environment, how UV ray stimulation will affect the flight behaviors of moths. The *Control Learner* is used to learn, in order to make a moth to exhibit specific flight behaviors, which UV ray stimulation should be performed. The output stimuli of the *Control Learner* are input to the *Behavior Learner* to generate expected flight parameters or input to real cyborgs to generate actual flight parameters, whose differences from the required flight parameters are used as feedback to tune the *Control Learner*.

The inputs to the *Behavior Learner* have the following three parts:

- The UV ray stimulation, which is described by 32 variables, i.e., the light intensities, exposure durations, pattern moving velocities (in the *x*-, *y*-, and *z*-axes), and pattern moving distances (in the *x*-, *y*-, and *z*-axes) of the four lamps.
- The ambient conditions, which are described by 34 variables, i.e., temperature, humidity, atmospheric pressure, oxygen concentration, carbon dioxide concentration, horizontal and vertical wind speeds, and the light duration, intensity, and illuminance of nine different wavelengths/colors (UVA, UVB, UVC, violet, indigo, blue, yellow, green, orange) to which the insect is sensitive (note that our study assumes that the ambient wind speed does not exceed 1.5 m/s; if the wind is too strong, then it is impossible to control the flight path of a moth).
- The previous flight parameter values of the moth, including seven values at the previous time step, and seven values accumulated over the previous three time steps using a memory neural network [25,26]. In this study, we set the interval between two time steps to 200 ms.

Therefore, the *Behavior Learner* takes 80 input variables to predict seven output flight parameter values. To address the complexity, instability, and uncertainty of moth flight behaviors, we divide the learner into three hierarchies from bottom to top: (1) the lower hierarchy of common layers, (2) the middle hierarchy of group-specific layers, and (3) the upper hierarchy of individual-specific layers.

The *Control Learner* takes inputs including (1) the user-specified (required) flight parameter values, (2) the ambient conditions, (3) the previous flight parameter values of the moth, and (4) the grouping information about the moth to output the UV ray stimulus values that are expected to make the moth perform the required flight behaviors (specified by the required flight parameter values).

The *Behavior Learner* aims to minimize the difference between the model output values and the actual flight parameter values of the cyborg moths. The *Control Learner* aims to minimize the difference between the required flight parameter values and the actual flight parameter values of the moths (or the output parameter values of the *Behavior Learner*) under the output stimuli of the *Control Learner*. We propose a Pythagorean fuzzy deep denoising autoencoder (PFDDAE) model to implement both the *Behavior Learner* and *Control Learner*.

### 2.2. Pythagorean Fuzzy Deep Denoising Autoencoder

In our approach, both the *Behavior Learner* and *Control Learner* use a denoising autoencoder (DAE) [27] as the basic building block. DAE is an autoencoder (AE) variant [28] that consists of an encoder and a decoder. The encoder transforms a *D*-dimensional input vector $x\in {\left[0,1\right]}^{D}$ into a hidden representation $y\in {\left[0,1\right]}^{D^{\prime }}$ through an affine mapping:

(1) f(x)=s(Wx+b)

where *s* is an activation function (typically an exponential or sigmoid function), W is a $D^{\prime }\;\times \;D$ weight matrix, and b is a $D^{\prime }$-dimensional bias vector (typically, we have $D^{\prime }\;<\;D$).

The decoder maps the hidden representation y back to a reconstructed vector $x^{\prime }$ in the input space with appropriately sized parameters $W^{T}$ and $b^{\prime }$:

(2) $$f^{\prime }\left(y\right)=s\left(W^{T}y+b^{\prime }\right)$$

AE learning determines appropriate parameters $\theta =\{W,b,b^{\prime }\}$ to minimize the reconstruction error (e.g., the squared error) over the training set T:

(3) $$\min J\left(\theta \right)=\frac{1}{\left|T\right|}\sum_{x\in T}L\left(x,x^{\prime }\right)$$

To improve the robustness to noise, DAE enhances the basic AE by corrupting any initial input x into $\tilde{x}$ by means of a stochastic mapping x into $q_{T}\left(\tilde{x}|x\right)$ and then maps the corrupted input $\tilde{x}$, as with the basic AE, to a hidden representation y from which we reconstruct an $x^{\prime }=f^{\prime }\left(y\right)$.

To capture the highly uncertain relationships between external stimuli and the species behaviors, we further enhance DAE by expressing the model parameters as fuzzy numbers, such that the model can effectively learn the fuzzy probability distribution over cross-layer units [29]. In particular, we employ Pythagorean-type fuzzy numbers (PFN) characterized by both a membership degree and a non-membership degree whose square sum is less than or equal to 1 [30,31] so as to not only allow for a larger body of membership grades than regular and intuitionistic fuzzy numbers but also enable each neuron to learn both how an input *contributes to* and how it *does not contribute to* the production of the output [32,33]. In this study, we use interval-valued PFN and use exp(·) as the activation function in Equation (1), where the exponential operation on fuzzy numbers is defined as in [34,35].

Nevertheless, using fuzzy model parameters makes the problem with the objective of minimizing Equation (3) a fuzzy optimization problem. Here, we utilize a centroid method [29,36] to defuzzify the problem. Given a PFN $\beta =P(\mu_{\beta },\nu_{\beta })$, its centroid point can be calculated as

(4) $$\begin{array}{c} c\left(\beta \right)=\left(c_{\mu }\left(\beta \right),c_{\nu }\left(\beta \right)\right)=\left(\frac{\int x\mu_{\beta }\left(x\right)dx}{\int \mu_{\beta }\left(x\right)dx},\frac{\int x\nu_{\beta }\left(x\right)dx}{\int \nu_{\beta }\left(x\right)dx}\right) \end{array}$$

Then, the distance between a crisp number α and a PFN β is calculated as

(5) $$\left|\alpha ,\beta \right|=\sqrt{{\left(c_{\mu }\left(\beta \right)-\frac{\alpha }{2}\right)}^{2}+c_{\nu }^{2}\left(\beta \right)}$$

Accordingly, for a Pythagorean-type fuzzy DAE, the reconstruction error of a fuzzy vector ${\tilde{x}}^{\prime }$ from a crisp vector x (with the same dimension *D*) is calculated as

(6) $$L\left(x,{\tilde{x}}^{\prime }\right)=\sqrt{\frac{\sum_{d=1}^{D}{\left|x_{d},{\tilde{x}}_{d}^{\prime }\right|}^{2}}{D}}$$

which is used in the objective function (3).

As a deep DAE is constructed by stacking layers of DAE [37], we construct a PFDDAE by stacking layers of Pythagorean-type fuzzy DAE, where each layer captures the hidden representation of the layer below as inputs, so as to effectively learn higher-order abstract representations from original input features.

Using fuzzy parameters can effectively improve the representation ability and robustness of the deep learning model [32,38], but it also increases the dimensionality of the problem, for which traditional gradient-based algorithms easily become trapped in local optima [39,40]. To improve the learning performance, we employ an evolutionary algorithm, water wave optimization (WWO) [41], to train the PFDDAE by evolving a population of solutions to explore the search space, making it more capable of jumping out of local optima. The algorithm first randomly initializes a population of solutions, each of which is a vector of network parameters and is evaluated by the reconstruction error of the corresponding model instance. The algorithm then continually evolves the solutions using WWO operators including propagation, refraction, and breaking [41]. The performance of WWO in training deep neural networks has been demonstrated by comparison with other state-of-the-art algorithms [42].

## 3. Behavior Leaner

### 3.1. Hierarchical Learning of Cyborg Flight Behaviors

To reduce the complexity of learning cyborg flight behaviors, we divide the *Behavior Learner* into three hierarchies, which are all implemented with PFDDAE.

#### 3.1.1. Learning the Species Common Behaviors

The lower hierarchy of layers is a PFDDAE for learning the common behaviors of the species, using unsupervised pretraining layer-by-layer to minimize the reconstruction error (3) of the PFDDAE.

#### 3.1.2. Learning Group-Specific Behaviors

A moth often exhibits stress behaviors similar to some other moths under the same environment. Grouping moths with similar behaviors and then learning group-specific behaviors can significantly improve the efficiency of model learning. However, there is no effective biological classification method for this purpose [43]. Here, we propose an improved fuzzy clustering method (described in the next subsection), which groups all moths into *c* groups and calculates a membership degree $u_{ij}$ of each *j*th moth to the *i*th group.

The middle group-specific hierarchy consists of a set of PFDDAE instances, each for learning the specific behaviors of a group of moths. Each group-specific learner takes the topmost representation of the lower common hierarchy as inputs and uses unsupervised pretraining to minimize the reconstruction error. However, here, the reconstruction error is weighted by fuzzy memberships, and the objective function for pretraining the *i*th group-specific learner is:

(7) $$\min J^{\left(i\right)}\left(\theta \right)=\frac{1}{\left|T\right|}\sum_{x_{j}\in T}u_{ij}L\left(x_{j},x_{j}^{\prime }\right)$$

Consequently, the larger the membership degree of a moth (input vector) $x_{j}$ to a group is, the higher the contribution of its reconstruction error to the objective function.

Ideally, whenever $u_{ij}\;>\;0$, $x_{j}$ participates in the training of the *i*th group-specific learner. However, in practice, to improve the computational efficiency, we set a lower limit $u^{L}$ and only use $T_{i}=\left\{x_{j}|u_{ij}\;>\;u^{L}\right\}$ as the training set for the *i*th group:

(8) $$\min J^{\left(i\right)}\left(\theta \right)=\frac{1}{|T_{i}|}\sum_{x_{j}\in T_{i}}u_{ij}L\left(x_{j},x_{j}^{\prime }\right)$$

#### 3.1.3. Learning Individual-Specific Behaviors

For each individual moth, we use a PFDDAE to learn its specific behaviors and utilize a multivariable linear regression (MLR) on the topmost individual-specific layer to produce the output flight parameter values. However, an individual-specific learner takes the outputs from multiple group-specific learners as its inputs. Let $D^{\prime \prime }$ be the output dimension of each group-specific learner; then, the input dimension of each individual-specific learner is also $D^{\prime \prime }$, and each input component $x_{d}^{\left(j\right)}$ of the *j*th individual-specific learner is calculated from multiple group-specific learners as follows ($1\;\leq \;d\;\leq \;D^{\prime \prime }$):

(9) $$x_{d}^{\left(j\right)}=\sum_{i=1}^{c}u_{ij}y_{d}^{\prime (i)}$$

where $y_{d}^{\prime (i)}$ denotes the *d*th component of the output vector of the *i*th group-specific learner. Consequently, the larger the membership degree $u_{ij}$ is, the higher the contribution of the output of the *i*th group-specific learner to the input of the *j*th individual-specific learner. Similar to the training set selection for a group-specific learner, in practice, we can only use the outputs from group-specific learners that satisfy $u_{ij}\;>\;u^{L}$ for training the *j*th individual-specific learner.

After pretraining an individual-specific learner according to the objective function (3) in an unsupervised manner, we train the learner in a supervised manner to minimize the regression error on the training set of the individual moth. However, since different flight parameters have different importance in determining the flight path, we use a weighted loss to evaluate the regression error:

(10) $$\min J_{R}^{\left(j\right)}\left(\theta \right)=\frac{1}{{\left|T\right|}^{\left(j\right)}}\sum_{x\in T^{\left(j\right)}}\sqrt{\sum_{d=1}^{7}w_{d}{\left(y_{d}^{\prime \prime }-{\hat{y}}_{d}^{\prime \prime }\right)}^{2}}$$

where $T^{\left(j\right)}$ denotes the set of labeled samples for the *j*th moth, $y_{d}^{\prime \prime }$ is the actual *d*th output component, ${\hat{y}}_{d}^{\prime \prime }$ is the expected *d*th output component, and $w_{d}$ is the weight importance of the *d*th output component. According to the statistics of the occurrences of different flight actions and contributions of different flight parameters to the actions (e.g., horizontal deflection occurs most frequently, and deflection angle is the most important parameter to the horizontal deflection action), we set the weight of the horizontal deflection angle to 0.24, the weight of the horizontal angular velocity to 0.2, the weight of the vertical deflection angle to 0.2, the weight of the vertical angular velocity to 0.15, and the weights of accelerations in the *x*-, *y*-, and *z*-axes to 0.07.

### 3.2. Pythagorean Fuzzy c-Means Clustering for Moth Grouping

We propose an improved fuzzy clustering method that uses moth shape information together with the outputs of the topmost common layer under three typical environmental settings combined with 12 typical UA ray stimulation settings to group moths.

As we know, fuzzy *c*-means clustering (FCM) [44] is a method for minimizing the overall fuzzy-membership-weighted distance of the data points from cluster centroids:

(11) $$\min J\left(U,V\right)=\frac{1}{cn}\sum_{i=1}^{c}\sum_{j=1}^{n}u_{ij}^{m}d_{ij}^{2}$$

where *n* is the number of data points, *c* is the number of clusters, $u_{ij}$ is the membership degree of the *j*th data point to the *i*th cluster subject to $\sum_{i}u_{ij}\;=\;1$, $d_{ij}$ is the distance between the *j*th data point and the *i*th cluster centroid, *m* is a control parameter larger than 1, $U={\left(u_{ij}\right)}_{c\;\times \;n}$ is the weight matrix, and $V\;=\;[v_{1},v_{2},\ldots ,v_{c}]$ is the vector of cluster centroids. Xu and Wu [45] extended the standard FCM to an intuitionistic FCM (IFCM) algorithm based on some new definitions of distance measures between intuitionistic fuzzy sets, which can capture more uncertainty information to improve clustering results.

We further improve the clustering method by using Pythagorean-type fuzzy sets to represent clusters. To group *D*-dimensional PFN data points, each cluster centroid is a *D*-dimensional PFN vector. The distance between two PFN $\beta_{1}=P\left(\mu_{\beta_{1}},\nu_{\beta_{1}}\right)$ and $\beta_{2}=P\left(\mu_{\beta_{2}},\nu_{\beta_{2}}\right)$ is defined as follows [46]:

(12) $$|\beta_{1},\beta_{2}|=\sqrt{\frac{{\left(\mu_{\beta_{1}}^{2}\;-\;\mu_{\beta_{2}}^{2}\right)}^{2}+{\left(\nu_{\beta_{1}}^{2}\;-\;\nu_{\beta_{2}}^{2}\right)}^{2}+{\left(\pi_{\beta_{1}}^{2}\;-\;\pi_{\beta_{2}}^{2}\right)}^{2}}{2}}$$

where $\pi_{\beta }=\sqrt{1\;-\;\mu_{\beta }^{2}\;-\;\nu_{\beta }^{2}}$ is the hesitant degree of β.

For any data point $x_{j}$ and cluster centroid $v_{i}$, we use the following distance measure to replace $d_{ij}$ in Equation (11):

(13) $$∥x_{j},v_{i}∥=\sqrt{\frac{\sum_{d=1}^{D}{\left|x_{jd},v_{id}\right|}^{2}}{D}}$$

Based on this new distance measure, we develop a Pythagorean FCM (PFCM) algorithm to cluster Pythagorean fuzzy sets. The pseudocode of the algorithm is shown in Algorithm 1 (where ϵ is a user-defined small positive value for controlling the stopping condition).

The performance of FCM clustering heavily depends on the quality of initial cluster centers [47,48]. Instead of randomly selecting the initial cluster centers, we also employ the WWO metaheuristic to search for optimal or high-quality initial cluster centers. Given the number *c* of clusters, WWO first randomly initializes a population of solutions, each of which represents a set $V^{\left(0\right)}$ of *c* initial cluster centroids and is evaluated by the objective function (11) of the clustering results derived from $V^{\left(0\right)}$. The algorithm then continually evolves the solutions until the stopping criterion is met.
**Algorithm 1:** Pythagorean fuzzy *c*-means clustering algorithm.
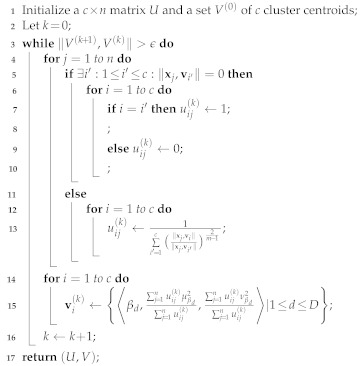


## 4. Control Leaner

The *Control Learner* learns which UV ray stimulus values can produce the required flight parameter values of a moth. It uses a PFDDAE to learn a high-order representation of the control mechanism, and it utilizes an MLR on top of the PFDDAE to output the recommended UV ray stimulus values. For a part of the training samples, we perform the output UV ray stimulus on physical moths and compare the actual flight parameter values exhibited by the moths with the required flight parameter values to evaluate the loss. However, because such physical experiments are costly, for a majority of training samples, we send the output UV ray stimulus to the *Behavior Learner* and compare the flight parameter values output by the *Behavior Learner* with the required flight parameter values to evaluate the loss. The two parts have different contributions to the final regression error. Let $T_{c}$ be the training set, $T_{p}$ be the subset whose outputs are sent to physical moths for comparison, and $T_{m}$ be the subset whose outputs are sent to the *Behavior Learner* for comparison. The regression error of the *Control Learner* is evaluated as

(14) $$\min J_{c}\left(\theta \right)=\frac{\sum_{x\in T_{p}}∥x,o_{p}\left(o_{c}\left(x\right)\right)∥+\;w_{m}\;\;\sum_{x\in T_{m}}∥x,o_{m}\left(o_{c}\left(x\right)\right)∥\;}{|T_{c}|}$$

where $o_{c}\left(x\right)$ is the *Control Learner*’s output stimulus vector according to input x, $o_{p}\left(o_{c}\left(x\right)\right)$ is the vector of flight parameter values of the physical moth under given stimuli $o_{c}\left(x\right)$, $o_{m}\left(o_{c}\left(x\right)\right)$ is the *Behavior Learner*’s output flight parameter values under given stimuli $o_{c}\left(x\right)$, and $w_{m}$ is a weight to emphasize the importance of physical verification. We set $w_{m}$ as inversely proportional to the average regression error of the *Behavior Learner* as follows (where each output component of the model is normalized into [0,1] in Equation (10)):

(15) $$w_{m}=1-\frac{\sum_{j}J_{R}^{\left(j\right)}\left(\theta \right)}{|T_{m}|}$$

## 5. Experiments

We conduct experiments to test (1) the performance of PFDDAE pretraining; (2) the performance of fuzzy clustering; (3) the performance of the PFDDAE-based *Behavior Learner*; and (4) the performance of PFDDAE-based *Control Learner*. The data set consists of 10,932 flight records of 36 moths, which were collected in a laboratory environment under different ambient conditions. Each data tuple consists of input UV ray stimuli and output flight parameters (as introduced in Section 2.1) for training the *Behavior Learner*; after training the *Behavior Learner*, the flight parameters are used as inputs to the *Control Learner*, and its output stimuli are input to the *Behavior Learner* or real cyborgs to produce expected/actual flight parameters in order to minimize their differences from the input flight parameters so as to train the *Control Learner*. The algorithms are executed on a workstation with an i7-6500 2.5 GH CPU, 8 GB DDR4 RAM, and an NVIDIA Quadro M500M card (Leadtek Research, Inc., Taiwan, China).

### 5.1. Experiments on Model Pretraining

We compare the following different algorithms, including gradient-based methods and evolutionary algorithms, for PFDDAE unsupervised pretraining:

- The basic gradient-based layer-wise (GLW) algorithm [28].
- An adaptive gradient (AdaGrad) algorithm [49].
- A non-revisiting genetic algorithm with adaptive mutation (NrGA) [50].
- A comprehensive learning PSO (CLPSO) algorithm [51] where each solution learns from different exemplars at different dimensions.
- A self-adaptive differential evolution (SaDE) algorithm [52], which adaptively chooses more prospective evolution strategies among a set of candidate strategies.
- A biogeography-based optimization (BBO) algorithm [53], which evolves solutions by continuously migrating features from high-fitness individuals to low-fitness ones based on a biogeographical migration model.
- An improved BBO algorithm called ecogeography-based optimization (EBO) [54], which defines two migration operators, namely global migration and local migration, that are adaptively applied according to the maturity of the population.
- The WWO algorithm [42].

On the data set, we tune the number of layers of each PFDDAE-based learner between two and five, and we find that it is sufficiently good to use three layers for all sub-learners of the *Behavior Learner* and use four layers for the *Control Learner*. We then tune the number of neurons of each layer between the square root and one-half of that of the previous layer. As a result, we set the model structure as follows:

- The three layers of the lower common learner have 80, 46, and 26 neurons, respectively;
- The three layers of each group-specific learner have 26, 15, and 9 neurons, respectively;
- The three layers of each individual-specific learner have 9, 6, and 7 neurons, respectively;
- The four layers of the *Control Learner* have 75, 49, 35, and 32 neurons, respectively.

To avoid overfitting, we conduct a five-fold cross-validation, i.e., the dataset is partitioned into five equal-sized pieces, and the validation is run five times, each using four pieces as the training set and using the remaining piece as the test set. A validation runs each algorithm 20 times with different random seeds. For the six evolutionary algorithms, the stopping criterion is set to that the number of objective function evaluations reaches 100,000 to ensure a fair comparison.

**Figure 3:**
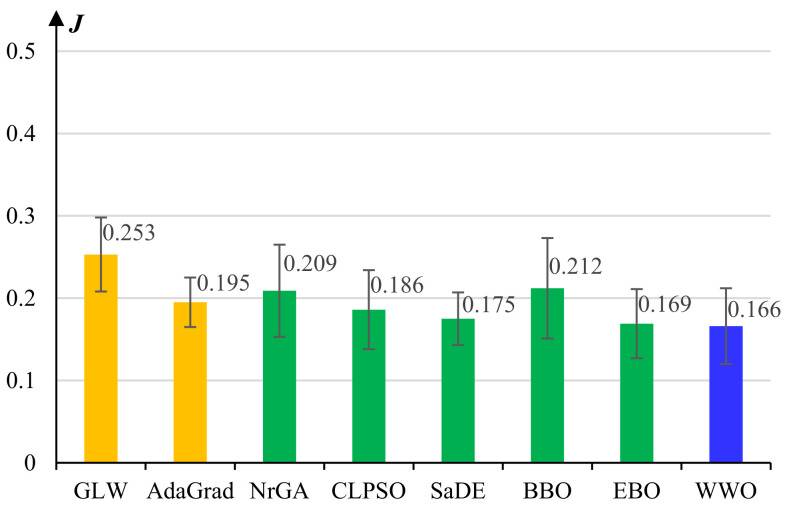
Average reconstruction errors and standard deviations of the comparative algorithms for pretraining the lower *common* layers of the *Behavior Learner*.

Figure 3 presents the average reconstruction errors and standard deviations of the comparative algorithms for pretraining the lower common learner of the *Behavior Learner*. The basic GLW algorithm exhibits the worst performance. All seven evolutionary learning algorithms achieve significant performance improvement over GLW, which demonstrates that population-based evolutionary algorithms can efficiently explore the high-dimensional solution space and therefore effectively overcome premature convergence. Nevertheless, not all evolutionary algorithms can outperform AdaGrad, which is another gradient-based algorithm. This indicates that to achieve promising training performance, we need to carefully design or adapt the evolutionary algorithms for this high-dimensional optimization problem. Among all eight algorithms, the proposed WWO algorithm obtains the lowest average reconstruction error, which evidences the efficiency of the nature-inspired metaheuristic for this complex optimization problem.

**Figure 4:**
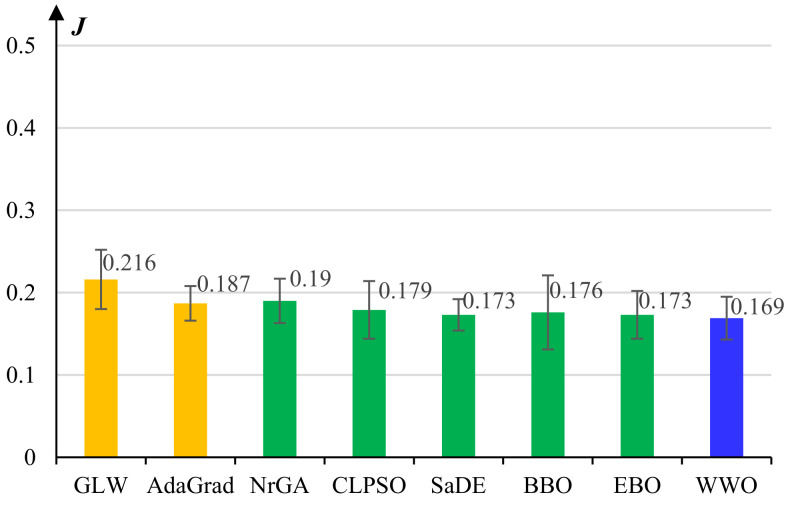
Average reconstruction errors and standard deviations of the comparative algorithms for pretraining the middle *group-specific* layers of the *Behavior Learner*.

Figure 4 presents the experimental results of the comparative algorithms for pretraining the middle group-specific learners. Similar to the experiments on the lower learner, GLW exhibits the worst performance and WWO exhibits the best performance. However, the performance advantages of WWO over the other evolutionary algorithms become less significant, because the number of neurons of a group-specific learner is nearly one-third that of the lower common learner, and hence, the problem dimension becomes much smaller.

**Figure 5:**
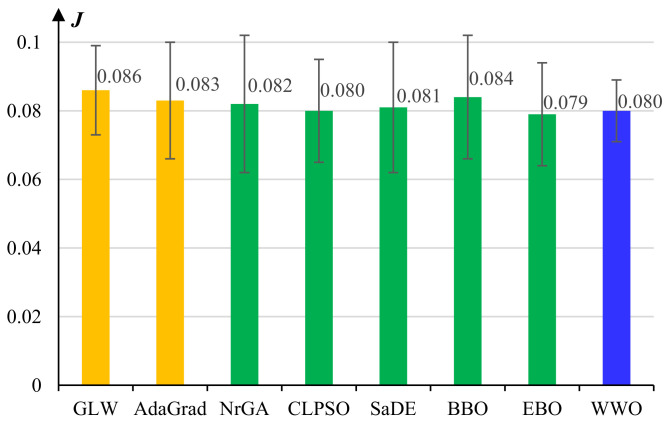
Average reconstruction errors and standard deviations of the comparative algorithms for pretraining the upper *individual-specific* layers of the *Behavior Learner*.

Figure 5 presents the experimental results of the comparative algorithms for pretraining the upper individual-specific learners. The number of neurons of an individual-specific learner is less than half that of a group-specific learner, and the problem dimension becomes even smaller. In this experimental section, except for the GLW that still exhibits the worst performance, the performance differences among the other algorithms are not very obvious. Consequently, for the *Behavior Learner*, we use WWO to pretrain the lower common layers and the middle group-specific layers, but employ AdaGrad to pretrain upper individual-specific layers—although most of the evolutionary learning algorithms exhibit slight performance advantages, AdaGrad consumes considerably fewer computational resources, especially when the number of individual moths is large.

**Figure 6:**
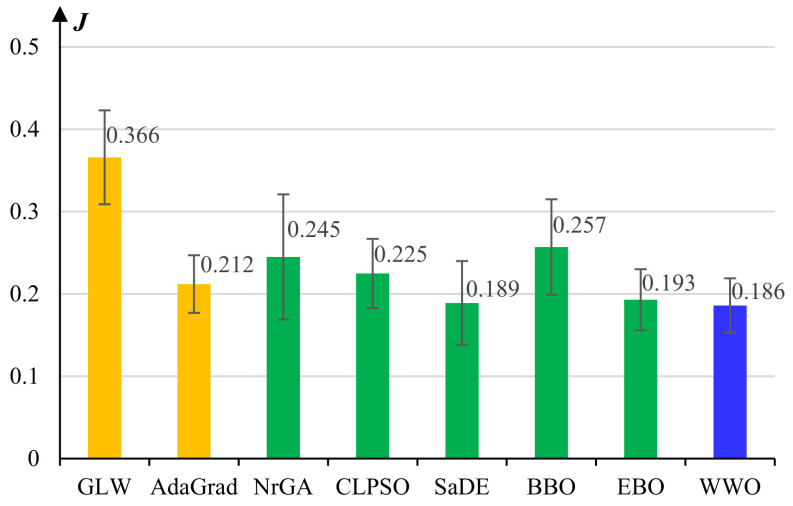
Average reconstruction errors and standard deviations of the comparative algorithms for pretraining the *Control Learner*.

Figure 6 presents the experimental results of the comparative algorithms for pretraining the *Control Learner*. For this high-dimensional optimization problem, we observe that the performances of some algorithms, including GLW, NrGA, and BBO, decrease significantly, while SaDE, EBO, and WWO still achieve high learning performances, and WWO obtains the lowest average reconstruction error among all the comparative algorithms. In general, the experimental results on model pretraining show that the larger the problem dimension is, the more significant the performance advantages of the well-designed evolutionary learning algorithms (especially the proposed WWO) over the classical learning algorithms.

### 5.2. Experiments on Fuzzy c-Means Clustering

We compare different clustering algorithms, including the basic FCM, IFCM, PFCM, and PFCM enhanced by evolutionary algorithms including NrGA [50], CLPSO [51], SaDE [52], BBO [53], EBO [54], and WWO [41]. After tuning the control parameters of the model on the data set, we set the number of clusters to five (too many clusters would consume a lot of computational resources, while fewer clusters cannot effectively discriminate individual moths and appropriately define group behaviors) and set m=2. We run each algorithm 20 times on the clustering problem.

**Figure 7:**
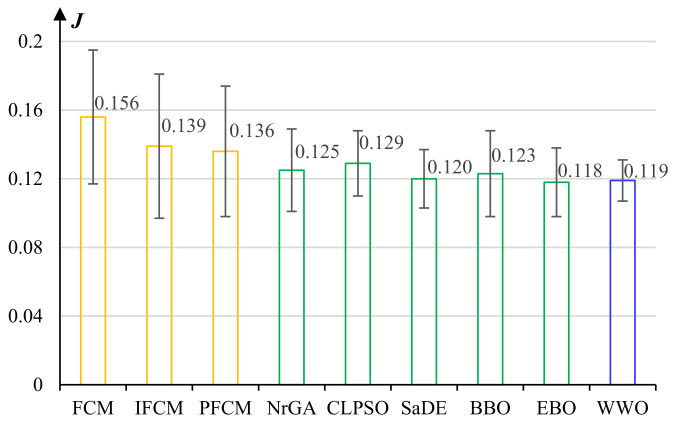
Average J(U,V) values and standard deviations of the clustering results of the comparative algorithms for moth grouping.

Figure 7 presents the average J(U,V) value of the clustering results of each comparative algorithm. By using intuitionistic fuzzy sets that have more expression ability than basic fuzzy sets, IFCM yields significantly higher clustering cohesion than the basic FCM. Pythagorean fuzzy sets further enlarger the body of membership grades, and PFCM exhibits higher clustering performance than IFCM.

FCM, IFCM, and PFCM all exhibit large standard deviations, i.e., their clustering results are quite unstable, because they use random cluster centroids. In comparison, evolutionary algorithms significantly improve the performance of PFCM by evolutionary searching for the optimal setting of cluster centroids. Among the six evolutionary algorithms, EBO obtains the best average J(U,V) value and WWO ranks second, but the standard deviation of WWO is much less than that of EBO. According to paired *t*-tests, there are no statistically significant differences among SaDE, BBO, EBO, and WWO. We select WWO because of its relatively high accuracy and robustness in clustering.

### 5.3. Experiments on Cyborg Flight Behavior Learning

After validating the performance of model pretraining and fuzzy clustering, we test the performance of the entire *Behavior Learner* for cyborg flight parameter prediction. The following models are implemented for comparison with the proposed PFDDAE model.

- A standard three-layer back-propagation artificial neural network (ANN). The numbers of neurons in the three layers are tuned to 80, 25, and 7, respectively.
- A basic self-adaptive neuro-fuzzy inference system (SANFIS) trained by an agglomerative clustering algorithm and a recursive least-squares algorithm [55].
- An evolving interval type-2 neuro-fuzzy inference system (IT2FIS) trained by a metacognitive sequential learning algorithm [56].
- A basic and integrated deep AE model (denoted by D-AE) [57], which employs the basic AE without a denoising mechanism as the building block and does not hierarchically divide the model into common learner, group-specific learners and individual-specific learners. After fine-tuning, the number of layers of D-AE is set to five, and the numbers of neurons in the three hidden layers are set to 48, 27, and 13, respectively.
- A basic and integrated deep DAE model (denoted by DDAE) [37], which uses the same structure as D-AE but employs DAE as the building block.
- A basic hierarchical deep DAE (denoted by HDDAE) that uses three hierarchies as described in Section 2 but does not employ fuzzy model parameters.
- A hierarchical fuzzy deep DAE (denoted by FDDAE) that uses three hierarchies as described in Section 2 but employs regular fuzzy numbers to represent model parameters [29].
- Three variants of the proposed model, which employs the *k*-means clustering (denoted by PFDDAE-kc), basic FCM [44] (denoted by PFDDAE-fc), and IFCM [45] (denoted by PFDDAE-ifc) instead of PFCM for moth grouping, respectively.

**Figure 8:**
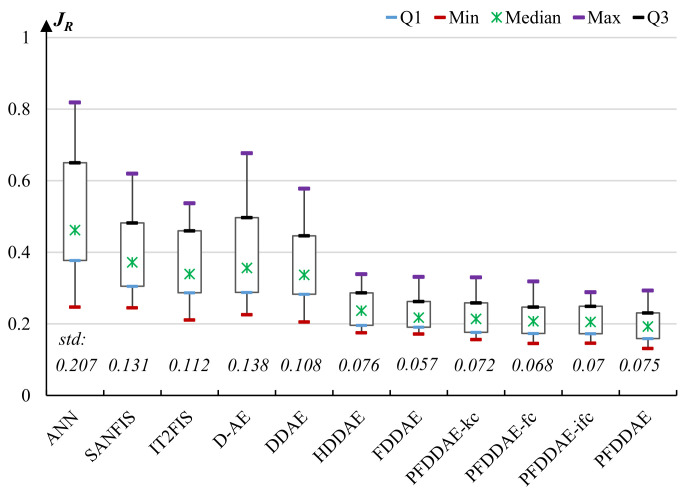
The performance (in terms of regression error) of the comparative models for cyborg flight behavior learning.

Figure 8 presents the box plots of the test results in terms of the objective function (10) obtained by the comparative models on the data set. The ANN has the highest error rate (46% on average) and instability, indicating that the shallow learning model is very ineffective for the complex flight parameter learning problem. The error rates of other models are much lower than that of ANN, which demonstrates that deep learning models can significantly improve learning performance by using multiple layers to discover intermediate representations. The average error rates of SANFIS, IT2FIS, D-AE, and DDAE are 37.2%, 33.9%, 35.6%, and 33.7%, respectively. Paired *t*-test results show that DDAE has significant performance improvement over SANFIS and D-AE, because inputs to the model typically contain much noise, and the denoising mechanism of DAE can reduce the effect of background noise. However, their error rates are still high and are unacceptable for engineering applications.

By dividing the deep learning model into three hierarchies for gradually learning the species’ common behaviors, group-specific behaviors, and individual-specific behaviors, our hierarchical deep learning model can effectively divide and conquer the model complexity and thus achieve significant performance improvement over not only the shallow ANN but also the monolithic neuro-fuzzy inference models and deep DAE models.

HDDAE, FDDAE, and PFDDAE-fc use the same hierarchical structure and FCM clustering method. According to paired *t*-tests, the results of FDDAE are significantly better than those of HDDAE, and the results of PFDDAE-fc are significantly better than those of FDDAE. This demonstrates that compared with using crisp model parameters, using fuzzy parameters can effectively improve the model’s representation ability and robustness; moreover, compared with using regular fuzzy parameters, using Pythagorean-type fuzzy parameters can further improve the learning performance by enabling each neuron to learn the contribution of input features to the output from both the positive and negative sides.

Comparing the results of the last four PFDDAE models, we also observe that the PFDDAE-fc model using the standard FCM clustering outperforms the PFDDAE-kc model using *k*-means clustering, and using intuitionistic and Pythagorean fuzzy sets can generate more valuable information for improving moth grouping and hence achieve better learning performance. Our PFDDAE model achieves an average error rate of 19.3%, which is the lowest among the 11 comparative models. The results demonstrate the performance advantages of the proposed PFDDAE using PFN parameters combined with PFCM clustering in learning cyborg behaviors.

### 5.4. Experiments on Cyborg Flight Control

Finally, we compare the performance of the PFDDAE-based *Control Learner* with other models including a fuzzy proportional–integral–derivative (F-PID) controller optimized by GA [58], ANN, an ANN with a filter (denoted by I-ANN) [59], a fuzzy nonlinear internal model control (FNIMC) with a robustness filter [60], SANFIS [55], IT2FIS [56], D-AE [57], DDAE [37], and FDDAE for cyborg flight control. The experiments use 20 moths and test 200 instructions for each moth. The performance is evaluated in two aspects. The first is the mean value of the objective function (14) obtained by each algorithm. The second is the success rate (SR) of flight instructions produced by each algorithm in actual moth flight control. We consider an instruction to be successful if, for each relevant flight parameter, the deviation of the actual output value from the expected value is less than 15%.

**Figure 9:**
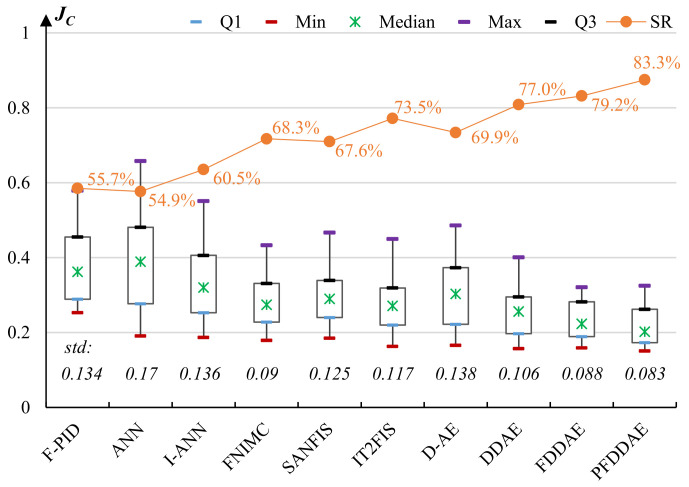
The performance (in terms of regression error and success rate) of the comparative models for cyborg flight control.

Figure 9 presents the box plots of the test results in terms of the objective function (14) and SR obtained by the comparative models. The traditional fuzzy PID and shallow ANN obtain high average error rates (approximately 40%) and low success rates (approximately 50%), indicating that they are not suitable for this difficult control problem. Compared to the simple ANN, ANN with a filter obtains a significantly lower error rate and higher success rate, but its performance is still much worse than the adaptive neuro-fuzzy models and deep learning models. The error rates of FNIMC, SANFIS, IT2FIS, and D-AE are around 27–30%, and their success rates are approximately 70%. By equipping D-AE with the denoising mechanism, DDAE decreases the average error rate to 25% and increases the success rate to 77%, which again demonstrates the importance of denoising in this sensitive control problem under complex environments. The error rates of FDDAE and PFDDAE are approximately 22% and 20%, and their success rates are over 79% and 83%, respectively, demonstrating that expressing model parameters with fuzzy numbers (in particular PFN) effectively enhances the model’s representation ability and robustness to improve control performance.

From the test results, we can also observe that for each model, the average error rate is inversely proportional to the success rate, which demonstrates the reasonability and practicability of the objective function of the control problem. In summary, the experiments show that the proposed PFDDAE exhibits both the best behavior learning performance and the best control performance among all comparative models, and its high success rate of over 83% indicates that it is suitable for this difficult cyborg flight control problem.

## 6. Conclusions and Discussion

In this paper, we present a fuzzy deep learning approach to the flight control of cyborg moths stimulated by UV rays. We propose a PFDDAE model to capture the highly uncertain relationships between external stimuli and the species behaviors. The PFDDAE model is employed for both the *Behavior Learner* for learning the flight behaviors of moths and the *Control Learner* for learning which UV ray stimulation can cause moths to exhibit required flight behaviors. To reduce complexity, the *Behavior Learner* is further divided into three hierarchies for learning the species’ common behaviors, group-specific behaviors, and individual-specific behaviors. The independent unsupervised pretraining of these parts significantly simplifies the task of model learning. We also propose the PFCM clustering method to group moths for learning group-specific behaviors. Experimental results demonstrate the performance advantages of the proposed approach, which obtains the lowest average error rate of 19.3% among the 11 comparative models for training the *Behavior Learner*, the lowest average error rate of 20% among the ten comparative models for training the *Control Learner*, and a high control success rate of over 83% that can provide sufficiently accurate control of the flight parameters of moths.

Cyborg insects have numerous potential applications. Our ongoing work includes studying solution methods for optimally controlling a cyborg moth as well as a swarm of moths to perform specific tasks such as path planning, surveillance and detection, and search and rescue [61,62]. For such complex control optimization problems, nature-inspired evolutionary algorithms are good alternatives to classical mathematical methods. We also plan to extend our cyborg moth control learning model to other cyborg insects, for which domain adaption and transfer learning methods [63] are expected to be useful.

## Abbreviations

The following abbreviations are used in this manuscript:

AdaGrad	Adaptive gradient
AE	Autoencoder
ANN	Artificial neural network
BBO	Biogeography-based optimization
DAE	Denoising autoencoder
DDAE	Deep denoising autoencoder
EBO	Ecogeography-based optimization
FCM	Fuzzy *c*-means clustering
GLW	Gradient-based layer-wise
IT2FIS	Interval type-2 neuro-fuzzy inference system
LED	Light-emitting diode
MLR	Multivariable linear regression
PFDDAE	Pythagorean fuzzy deep denoising autoencoder
PFN	Pythagorean-type fuzzy number
PSO	Particle swam optimization
SANFIS	Self-adaptive neuro-fuzzy inference system
UV	Ultraviolet
WWO	Water wave optimization
